# An efficient optimizer for the 0/1 knapsack problem using group counseling

**DOI:** 10.7717/peerj-cs.1315

**Published:** 2023-04-14

**Authors:** Yazeed Yasin Ghadi, Tamara AlShloul, Zahid Iqbal Nezami, Hamid Ali, Muhammad Asif, Hanan Aljuaid, Shahbaz Ahmad

**Affiliations:** 1 Department of Computer Science/Software Engineering, Al Ain University, Al Ain, UAE; 2 Collage of General Education, Liwa College of Technology, Abu Dhabi, UAE; 3 Department of Computer Science, The Superior University, Lahore, Pakistan; 4 Department of Computer Science, National Textile University, Faisalabad, Pakistan; 5 Computer Sciences Department, College of Computer and Information Sciences, Princess Nourah bint Abdulrahman University, Riyadh, Saudi Arabia

**Keywords:** GCO, Knapsack, Evolutionary algorithmx, Evolutionary algorithm, Combinatorial, Optimization, Machine learning

## Abstract

The field of optimization is concerned with determining the optimal solution to a problem. It refers to the mathematical loss or gain of a given objective function. Optimization must reduce the given problem’s losses and disadvantages while maximizing its earnings and benefits. We all want optimal or, at the very least, suboptimal answers because we all want to live a better life. Group counseling optimizer (GCO) is an emerging evolutionary algorithm that simulates the human behavior of counseling within a group for solving problems. GCO has been successfully applied to single and multi-objective optimization problems. The 0/1 knapsack problem is also a combinatorial problem in which we can select an item entirely or drop it to fill a knapsack so that the total weight of selected items is less than or equal to the knapsack size and the value of all items is as significant as possible. Dynamic programming solves the 0/1 knapsack problem optimally, but the time complexity of dynamic programming is O(n^3^). In this article, we provide a feature analysis of GCO parameters and use it to solve the 0/1 knapsack problem (KP) using GCO. The results show that the GCO-based approach efficiently solves the 0/1 knapsack problem; therefore, it is a viable alternative to solving the 0/1 knapsack problem.

## Introduction

Over the previous several decades, the use of evolutionary algorithms to solve optimization issues has increased. Single-objective and multi-objective are the two categories of optimization problems. Multi-objective optimization involves problems with multiple competing goals to be achieved. Problems that have only one goal are called single-objective optimization problems. Researchers have suggested several evolutionary optimization strategies for single-objective and multi-objective optimization problems (Steuer, 1986). These include the Adaptive neuro-fuzzy inference system-evolutionary algorithms hybrid models (ANFIS-EA) (Roy et al., 2020), multi-objective optimization of grid-connected PV-wind hybrid system (Barakat, Ibrahim & Elbaset, 2020), ant colony optimization (ACO) (Dorigo, Birattari & Thomas, 2006), evolution strategy (ES) (Mezura-Montes & Coello Coello, 2005), particle swarm optimization (PSO) (Janga Reddy & Nagesh Kumar, 2021; Coello Coello, Pulido & Lechuga, 2004), genetic algorithm (Deb et al., 2002), genetic programming (GP) (Mugambi & Hunter, 2003), evolutionary programming (EP) (Fonseca & Fleming, 1995), differential evolution (DE) (Storn & Price, 1995), group counseling optimizer (Eita & Fahmy, 2010; Ali & Khan, 2013), comprehensive parent selection-based genetic algorithm (CPSGA) (Ali & Khan, 2012), whale optimization algorithm (Masadeh, 2021), binary particle swarm optimization algorithm (Sun et al., 2021), hybrid cat-particle swarm optimization algorithm (Santoso et al., 2022), An enhanced binary slime mold algorithm (Abdollahzadeh et al., 2021), improving flower pollination algorithm (Basheer & Algamal, 2021), and 0/1 knapsack problem using genetic algorithm (Singh, 2011), are some of the most common evolutionary optimization techniques. The group counseling optimizer (GCO) ensures uniqueness to prevent premature convergence. The effectiveness of the GCO method demonstrates that it is well-suited to solving both single-objective and multi-objective optimization problems (Ali & Khan, 2013).

Since the 0/1 knapsack problem is a combinatorial problem, approaches such as branch and bound, backtracking, and dynamic programming are not very helpful in solving it. A multi-objective variant of GCO has been recently published that shows promise in solving multi-objective optimization problems. According to the results, group counseling optimizer is used in a real-world application to test its applicability and the proposed algorithm also outperforms well-known optimizers.

In this article, we use the GCO algorithm for the following:

1. to solve the 0/1 knapsack problem and
2. to analyze the various parameters to see how they affect the solution.

The experimental results show the significant role of parameters in GCO algorithms for solving the 0/1 knapsack problem.

The rest of the article is organized as follows: Section II describes the related work. In section III, we briefly discuss the group counseling optimizer. Sections IV and V describe the knapsack problem and experimental results, respectively. Section VI concludes the article.

## Related Work

In Ezugwu et al. (2019), the authors proposed preliminary results from several memetics optimization procedures for solving the 0/1 knapsack problems, including simulated annealing, genetic algorithms, greedy search algorithm, dynamic programming, branch and bound, and simulated annealing using a hybrid genetic algorithm. During the optimization process, every computation punishes infeasible arrangements while optimizing the feasible ones. The authors of Abdel-Basset, Mohamed & Mirjalili (2021) suggested a binary version of equilibrium optimization (BEO). Transfer functions change the constant value from continuous to binary EO, converting it to the binary values 0, 1, and 0. Transfer functions come in two different shapes: V-shaped and S-shaped. This research demonstrates that the best transfer function among them is the V-Shaped V3. The sigmoid S3 transfer function is likewise more advantageous than V3. This new intelligent system builds on dragonfly foraging and predator evasion theory (Wang, Shi & Dong, 2021). When tackling continuous multi-modal functions and engineering issues, the dragonfly algorithm (DA) excels. This study offers an angle modulation technique on DA (AMDA). AMDA is used to produce bit strings to make this algorithm function in binary space. A modified AMDA, an improved angle-modulated dragonfly algorithm (IAMDA) is presented to increase algorithm stability and convergence speed by including one additional coefficient to control the vertical displacement of the cosine section of the original generating function. Hybrid rice optimization (HRO) (Shu et al., 2022) is a new optimization procedure stimulated through the upbringing method of Chinese three-line hybrid rice. The population of the algorithm is divided into three categories; restorer, maintainer, and sterile line, and other stages like hybridization, renewal, and selfing are applied. The proposed algorithm is integrated with another to develop a parallel and serial model using the binary ant colony optimization (BACO) technique to expand merging speed and search efficiency. The maintainer line is updated by BACO, which is incorporated in HRO as an operator.

The re-establishment plan presents an active step to adjust the investigation and misuse stages. All exploratory approaches were implemented using Python 3.6 on a Windows 10 Working Framework PC with an Intel(R) Core (TM) i7-8700 @ 3.2 GHz CPU and 16GB DDR3 RAM. In Moradi, Kayvanfar & Rafiee (2021), the authors develop and contrast a novel population-based SA (PSA) for the 0/1 knapsack problem and analyze and contrast the given and non-introduced single-solution SA-based computations for KP01. PSA is the most efficient optimization strategy for KP01 among the SA-based options. PSA’s inquiry and abuse are far more capable than previous SA-based calculations since it generates multiple beginning arrangements rather than a single fair one. PSA employs transformation and hybrid administrators to investigate and misuse the arrangement space and the ravenous repair and improvement instrument to identify neighboring arrangements. In Abdollahzadeh et al. (2021), the authors solve the 0/1 knapsack problem using an extended binary slime mold algorithm (SMA) at various sizes. SMA has tested and analyzed eight distinct transfer functions. The transfer function that performed well is used to improve the performance of the proposed binary SMA, and the Bitwise and Gaussian mutation operators are applied. Punitive work and a repair computation are used to deal with infeasible arrangements. On typical datasets with various scales, performance was tested statistically. In Basheer & Algamal (2021), the authors proposed two new time-varying exchange functions to expand the biotic flower pollination algorithm BFPA’s investigation and exploitation capacity with the best arrangement and shortest computation time. A bio-inspired algorithm called the flower pollination algorithm mimics the pollination characteristics of plants’ blooms. It mimics the backpack issue’s small, medium, and large-dimensional scales. Two efficient time-varying exchange functions are combined with the S-shaped and V-shaped functions with the time-varying notion. In Wang & Wang (2021), the authors provide a quantum-inspired differential advancement algorithm with a grey wolf optimizer (QDGWO) to make strides in differences and meet execution in high-dimensional situations. The suggested method uses quantum computing standards such as quantum superposition states and gates. It, too, uses flexible transformation operations of differential advancement, hybrid operations of differential advancement, and quantum perception to construct unique arrangements as trial persons. The superior arrangements between the stored individuals and the trial individuals formed by change and hybrid operations are determined using determination operations.

## Group Counseling Optimizer

**Figure 1:**
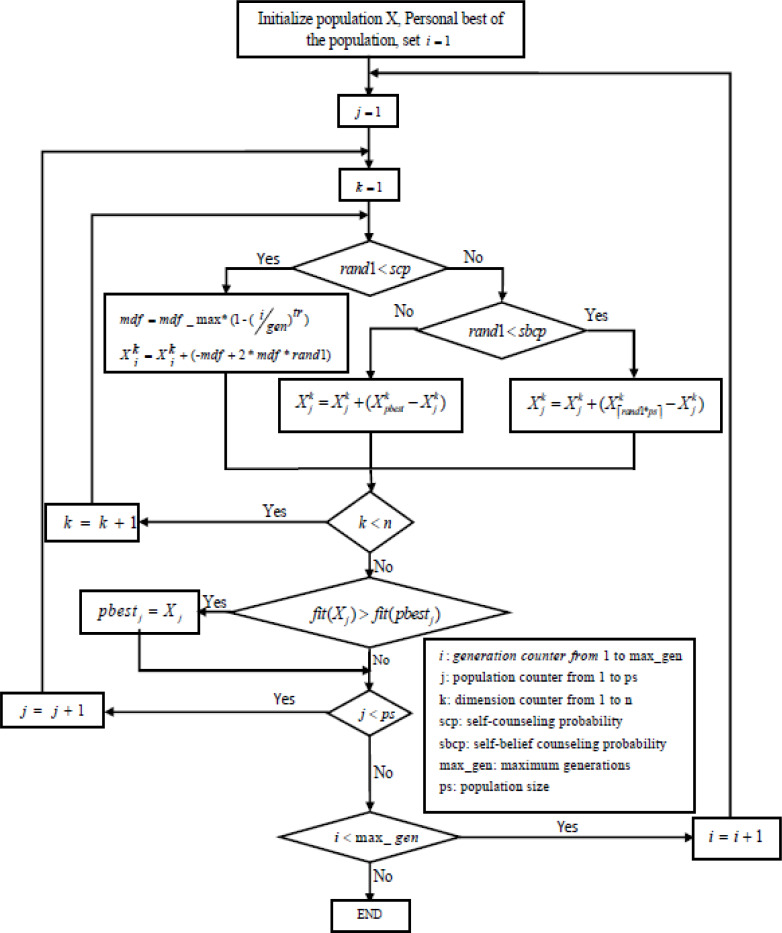
Flowchart of GCO algorithm.

As was previously said, the GCO draws inspiration from human behavior to solve real-world problems (Eita & Fahmy, 2010; Ali & Khan, 2013). Several parameters must be established, including the population size, generational age, generational size of the counselors, self-counseling probability, and self-belief counseling probability (SBCP). Figure 1 describes the flowchart of the GCO algorithm.

### Main algorithm

The outline of the GCO algorithm is given in algorithm 1:


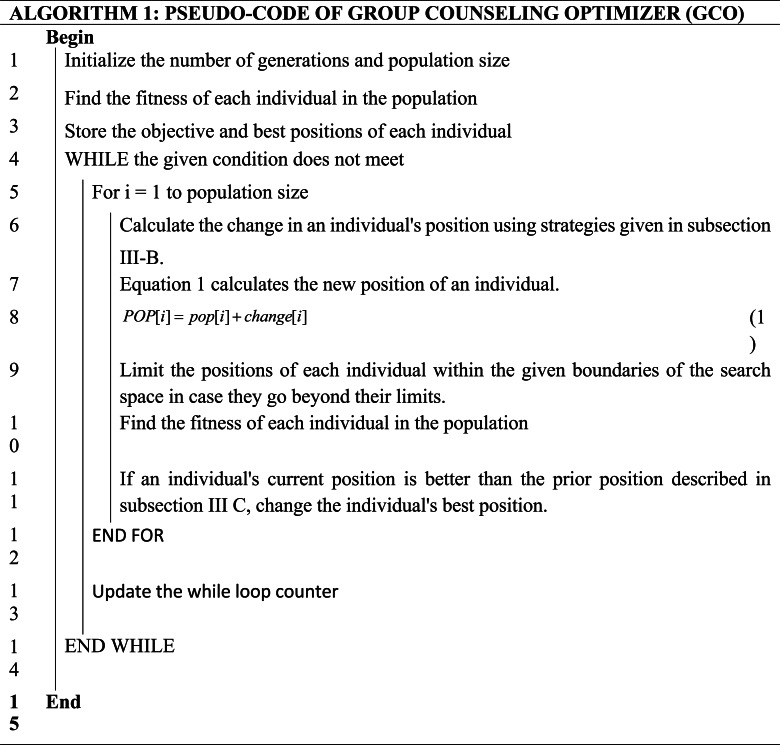


### Selection of individuals for counseling

The counseling process of each individual continues component-wise in each generation. The new value of each component is obtained from the previous values of the same component using one of the following three strategies:

• Other-members counseling
• Self-belief counseling (use self-best values)
• Self-counseling

To influence a person’s decision, the creators of the original GCO algorithm (Eita & Fahmy, 2010) provide other members with counseling and self-counseling techniques. However, in Ali & Khan (2013), the authors add a third technique called self-belief coaching to help an individual stay current.

Strategy, *i.e.,* self-belief counseling, illustrates an individual’s self-experience in decision-making as we know that an individual’s self-experience influences their decisions. This self-experience tries to limit any possible modification against what they believe based on their personal experience. Therefore, the authors introduce the self-belief counseling strategy into the algorithm to make the counseling process more realistic.

For each component, we generate a random number of self-counseling decisive coefficients (*sdc*) in the range [0, 1]. If “*sdc*” is less than self-counseling probability (*scp*), then we do self-counseling; else, we do self-belief or other-members counseling. Now we generate a random number self-belief counseling decisive coefficient “*scdc*” in the range [0, 1]. If “*scdc*” is less than the self-belief counseling probability (*scdc*), use the self-belief counseling strategy; otherwise, use other member counseling strategies. The self-counseling operator covers the whole range of each component at the start of the search and then uses a nonlinear function to limit the range covered over time. So, self–counseling produces a highly explorative behavior at the beginning of the search, and the influence of the self-counseling operator reduces as the number of generations grows.

The process of computing the new value of each component is given in algorithm 2.


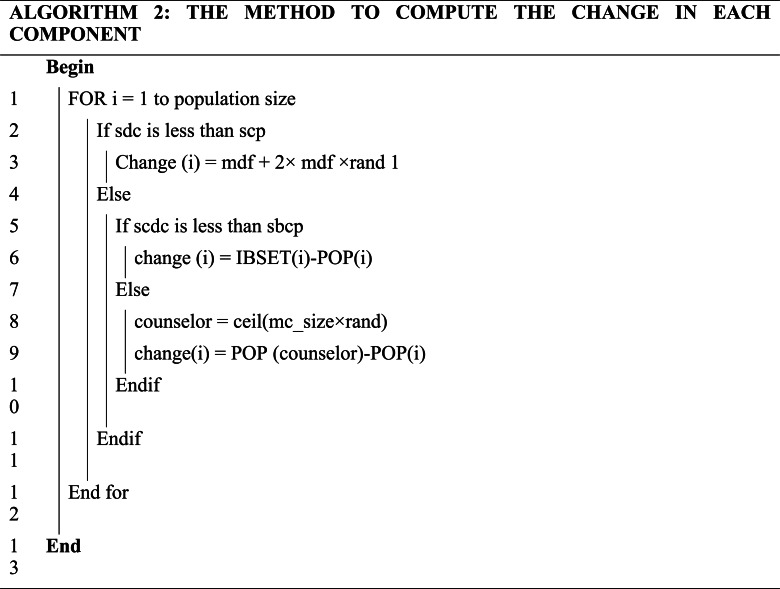


Where,

(1) \begin{eqnarray*}mdf=\max \nolimits \text{_}mdf\times (1- \frac{iteration}{gen} )^{tr}\end{eqnarray*}

### Updating individual best position

If the present position leads to the best value, replace the best position; otherwise, do not change the best position. If the current and best positions are the same, then we can randomly select any position.


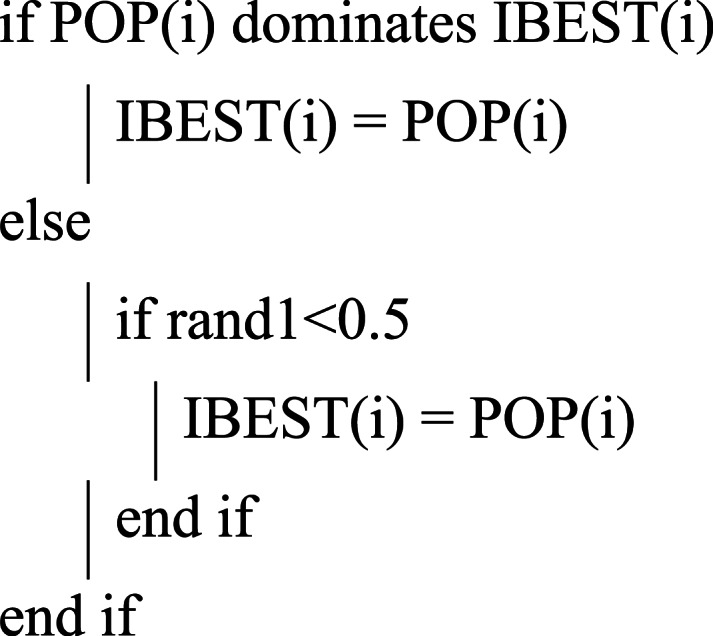


## Knapsack Problem

The 0/1 knapsack problem is a combinatorial problem. ‘*W*’ is the positive capacity of a knapsack. An individual can insert a set of *S* different items in the knapsack. The weight of item ‘*i*’ is a positive integer ‘wi’, while the value of item ‘*i*’ is a positive integer vi (Singh, 2011). The objective is to:

\begin{eqnarray*}\begin{array}{@{}l@{}} \displaystyle \text{Maximize}\\ \displaystyle \sum _{i=1}^{m}{v}_{i}{s}_{i}\\ \displaystyle subject~to\\ \displaystyle \sum _{i=1}^{m}{w}_{i}{s}_{i}\leq W \end{array} \end{eqnarray*}

### Example of 0/1 knapsack problem

Assume that there is a knapsack with a size of 15 and many objects of varying weights and values. Within the constraints of the knapsack’s capacity, we wish to maximize the worth of goods contained in the knapsack. Four objects were used (A, B, C, and D). The following are their weights and values as shown in Table 1:

**Table 1:** Detail of example knapsack problem items.

**Item #**	A	B	C	D
Values	25	35	40	55
Weights	8	6	7	9

We want to maximize the total value:

(2) \begin{eqnarray*}\sum _{i=1}^{4}({v}_{1}{s}_{1}+{v}_{2}{s}_{2}+{v}_{3}{s}_{3}+{v}_{4}{s}_{4}).\end{eqnarray*}

(3) \begin{eqnarray*}\begin{array}{@{}l@{}} \displaystyle \text{Maximize}\\ \displaystyle \sum _{i=1}^{4}{v}_{i}{s}_{i}=(25{s}_{1}+35{s}_{2}+40{s}_{3}+55{s}_{4}){\mathrm{s}}_{i}\in (0,1)\\ \displaystyle i=1,2,\ldots ,m\\ \displaystyle subject~to\\ \displaystyle \sum _{i=1}^{4}{w}_{i}{s}_{i}=(8{s}_{1}+6{s}_{2}+7{s}_{3}+9{s}_{4})\leq W \end{array}\end{eqnarray*}

There are 24 potential subsets of objects for this problem, as shown in Table 2:

**Table 2:** Possible subsets of items.

A	B	C	D	Total weight	Total value
0	0	0	0	0	0
0	0	0	1	9	55
0	0	1	0	7	40
0	0	1	1	16	95
0	1	0	0	6	35
0	1	0	1	15	90
0	1	1	0	13	75
0	1	1	1	22	130
1	0	0	0	8	25
1	0	0	1	17	80
1	0	1	0	15	65
1	0	1	1	24	120
1	1	0	0	14	60
1	1	0	1	23	115
1	1	1	0	21	100
1	1	1	1	30	155

The best solution satisfies the constraint while also providing the highest total value. The rows in italics face meeting the condition in our situation. As a result, the best value for the given constraint (*W* = 15) is 90, which is reached with B and D items.

## Results and Discussion

The item representation, individual encoding, experimental setup, and conclusions are discussed in this section.

### Representation of the items

To represent all items, we need a table with three rows, item #, weights, and values, as seen in Table 3:

**Table 3:** Representation of the item.

**Item #**	1	2	3	4
**Weights**	8	6	7	9
**Values**	25	35	40	55

### Encoding an individual

An array of the same size represents an individual as the number of elements in the array. The item included in the knapsack is denoted by 1; if not included, indicated by 0, but the total weight is always less than or equal to the knapsack size. For example, the second and fourth items are packed in the knapsack, as shown in Table 4.

A table called “population” is used to represent the entire population. The rows represent the number of individuals, and the columns represent the items that might be carried in the knapsack.

### Fitness function

Each individual’s fitness is equal to the sum of the values of selected items for the knapsack.

### Termination condition

**Figure 2:**
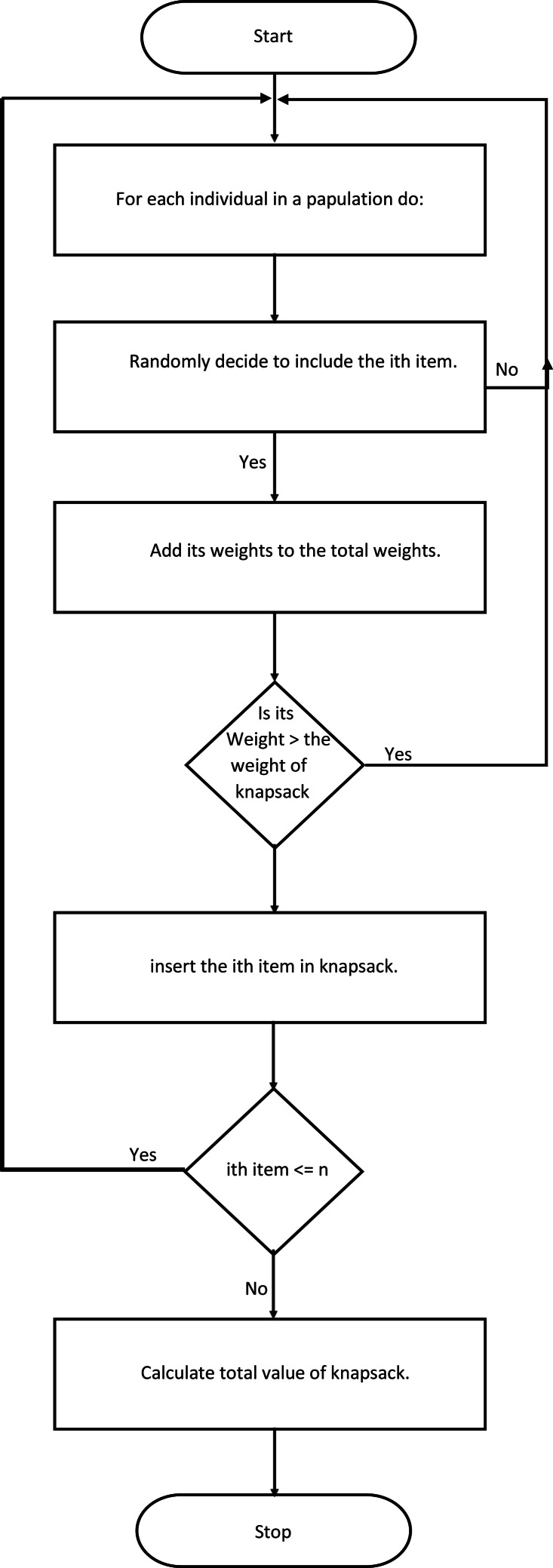
A flow chart for the fitness function.

After achieving the provided criteria (maximum number of generations or maximum assigned profit value), the program will terminate. The whole process is given in Fig. 2.

**Table 4:** Encoding of an individual.

**Item #**	1	2	3	4
Weights	0	1	0	1

### Experimental setup

MATLAB R2018a has been used to develop the GCO algorithm-based solution to solve the 0/1 knapsack problem. A thorough analysis was performed on the performance impact of the parameters used in the GCO algorithm. The three knapsack problems with different weights are given in Tables 5 to 7. These problems were used for various experiments.

**Experiment 1:** At the same time, we have changed the number of counselors. Analysis was performed by 1 to 5 counselors. The other settings were left alone and followed (Ali & Khan, 2013) exactly.

**Experiment 2:** The number of function evaluations was changed from 200 to 1,000, as given in Tables 8 to 10. We changed the population sizes to 10, 20, 30, 40, and 50 while keeping the number of generations equal to 100. The other settings were left alone and followed (Ali & Khan, 2013) exactly.

**Table 5:** A 0/1 knapsack problem with the weight of the knapsack = 60.

**Item #**	1	2	3	4	5	6	7	8	9	10
Values	35	30	27	25	20	18	17	14	13	10
Weights	15	12	10	13	11	9	8	7	5	4

**Table 6:** A 0/1 knapsack problem with the weight of the knapsack = 70.

**Item #**	1	2	3	4	5	6	7	8	9	10
Values	10	13	14	17	18	20	25	27	30	35
Weights	3	4	6	8	10	12	14	17	18	19

**Table 7:** A 0/1 knapsack problem with the weight of the knapsack = 80.

**Item #**	1	2	3	4	5	6	7	8	9	10
Values	25	23	28	29	37	34	36	39	40	42
Weights	6	9	12	14	17	19	20	15	18	20

**Table 8:** Impact of number of counselor for the first problem.

**Counselors →**	**1**	**2**	**3**	**4**	**5**
**Best**	140	140	140	140	140
**Worst**	133	133	135	133	126
**Average**	137	136	137	137	137
**Median**	137	137	137	137	137
**Std. Dev.**	2.20	2.29	2.16	2.27	2.66

**Table 9:** Impact of number of counselors for the second problem.

**Counselors →**	**1**	**2**	**3**	**4**	**5**
**Best**	139	139	139	139	139
**Worst**	126	129	124	127	124
**Average**	135	134	133	134	133
**Median**	136	134	134	135	133
**Std. Dev.**	2.98	2.54	2.97	2.63	3.00

**Table 10:** Impact of number of counselors for the third problem.

**Counselors →**	**1**	**2**	**3**	**4**	**5**
**Best**	197	197	197	197	197
**Worst**	166	169	168	176	169
**Average**	188	189	187	188	187
**Median**	188	189	187	188	188
**Std. Dev.**	5.40	4.74	5.71	4.26	5.91

**Experiment 3:** The number of function evaluations was changed from 200 to 1,000, as given in Tables 11 to 13. We changed the number of generations to 20, 40, 60, 80, and 100 while keeping the population size equal to 100. The other settings were left alone and followed (Ali & Khan, 2013) exactly.

**Table 11:** Impact of the number of function evaluations and number of population for the first problem.

**Counselors →**	**200**	**400**	**600**	**800**	**1000**
**Best**	140	140	140	140	140
**Worst**	130	135	135	135	137
**Average**	137	139	139	140	139
**Median**	137	140	140	140	140
**Std. Dev.**	2.39	1.59	1.21	0.9	0.4

**Table 12:** Impact of the number of function evaluations and number of population for the second problem.

**Counselors →**	**200**	**400**	**600**	**800**	**1000**
**Best**	139	139	139	139	139
**Worst**	124	131	131	131	134
**Average**	134	136	136	137	138
**Median**	133	136	136	137	138
**Std. Dev.**	3.12	2.34	2.00	1.66	1.27

**Table 13:** Impact of the number of function evaluations and number of population for the third problem.

**Counselors →**	**200**	**400**	**600**	**800**	**1000**
**Best**	140	140	140	140	140
**Worst**	130	135	134	135	133
**Average**	137	138	138	138	138
**Median**	137	140	140	140	140
**Std. Dev.**	2.38	2.22	2.17	2.30	2.36

**Experiment 4:** In this experiment, we changed the self-belief counseling probability. The results are produced by varying the self-belief counseling probability from 10% to 90%, as given in Tables 14 to 16. The other settings were left alone and followed (Ali & Khan, 2013) exactly.

**Table 14:** Impact of the number of the function evaluations and number of generations for the first problem.

**Counselors →**	**200**	**400**	**600**	**800**	**1000**
**Best**	197	197	197	197	197
**Worst**	176	178	187	184	187
**Average**	188	191	192	192	193
**Median**	188	192	193	193	194
**Std. Dev.**	4.42	3.68	2.71	2.96	2.41

**Experiment 5:** In this experiment, we changed the self-counseling probability. The results are produced by varying the self-counseling probability from 10% to 90%, as given in Tables 17 to 19. The other settings were left alone and followed (Ali & Khan, 2013) exactly.

**Table 15:** Impact of the number of function evaluations and number of generations for the second problem.

**Counselors →**	**200**	**400**	**600**	**800**	**1000**
**Best**	139	139	139	139	139
**Worst**	129	127	125	131	131
**Average**	134	136	136	136	137
**Median**	135	136	136	136	138
**Std. Dev.**	2.95	2.81	2.75	2.12	2.39

The following subsections explain the findings.

### Experiment 1

This experiment sought to determine how the GCO algorithm’s use of counselors affected its ability to solve the 0/1 knapsack problem. We compare the GCO results by varying the number of counselors using three different knapsack problems.

Tables 8 to 10 show that the average and standard deviation of results produced by varying the number of counselors are best when the number of counsellors is equal to 1 for all three knapsack problems. So we should use only one counselor for knapsack problems.

### Experiment 2

This experiment was designed to find the impact of the number of function evaluations and population size in the GCO algorithm when solving the 0/1 knapsack problem. We compare the GCO results by varying the number of function evaluations using three different knapsack problems.

Tables 11 to 13 show that the error decreases as the number of function evaluations increases. Because the average and standard deviation of results produced by varying the number of function evaluations are minima when the number of functions is equal to 1,000 for all three knapsack problems, we can say that GCO does not trap knapsack problems at a local optimum.

### Experiment 3

This experiment aimed to determine how the GCO algorithm’s function evaluations and generation count affected its ability to solve the 0/1 knapsack problem. We compare the GCO results by varying the number of function evaluations using three different knapsack problems.

Tables 14 to 16 show that the error decreases as the number of function evaluations increases. Because the average and standard deviation of results produced by varying the number of function evaluations are minima when functions are equal to 1,000 for all three knapsack problems, we can say that GCO is not a trap for knapsack problems at the local optimum.

Comparing the results produced in experiments 2 and 3 shows that increasing the number of generations is more effective than a population. So, we use more generations except for the population.

### Experiment 4

This experiment aimed to determine how the self-belief counseling probability affected the GCO algorithm’s capacity to solve the 0/1 knapsack problem. Compare the GCO results using three different knapsack problems and varying the probability of trust advice from 10% to 90%.

Tables 17 to 19 show that the average and standard deviation results produced by varying the self-belief-counseling probability are best at 70% for two out of three knapsack problems. So we should use a 70% self-belief-counseling probability for knapsack problems.

### Experiment 5

This experiment aimed to determine how the self-counseling probability affected the GCO algorithm’s capacity to solve the 0/1 knapsack problem. We compare the GCO results by varying the self-counseling probability from 10% to 90% using three knapsack problems.

Tables 20 to 22 show that the average and standard deviation of results produced by varying the self-counseling probability is best at 50% for two out of three knapsack problems. So we should use a 50% self-belief-counselling probability for knapsack problems.

**Table 16:** Impact of the number of function evaluations and number of generations for the third problem.

**Counselors →**	**200**	**400**	**600**	**800**	**1000**
**Best**	197	197	197	197	197
**Worst**	176	179	182	175	182
**Average**	188	190	190	191	191
**Median**	188	191	192	193	193
**Std. Dev.**	4.77	3.81	3.33	4.12	3.50

A proposed solution for the 0/1 knapsack problem based on GCO is implemented in MATLAB R2018a. GCO advanced parameter settings are initialized as follows:

• The population size is equal to 50;
• The maximum number of generations increases from 20 to 100;
• The number of counselors is equal to 1;
• self-belief-counseling probability is equal to 70%;
• self-counseling probability is equal to 50%;
• Max_mdf is equal to 0.5;

In all experiments, we report results obtained from 100 independent runs. The best results are presented in all cases.

### Results

Our experiments use the 0/1 knapsack problem with three different knapsack capacities. Details of the 0/1 knapsack problem are shown in Table 2 to Table 4.

Set the population size to 20 and vary the number of generations from 20 to 100 to generate results. Tables 5 to 7 show the maximum fit found after 100 runs of the algorithm in each generation. The results demonstrate the usefulness of GCO for the 0/1 knapsack problem. They show that for all numbers of function evaluations, it yields results close to those of the dynamic programming approach for all three problems.

Diff = ((Optimal value –GCO Optimal value)/ Optimal value) *100

Various problem sizes are present in the test scenarios to examine the effectiveness of the proposed algorithm. The numbers of items that sizes are 20, 40, 60, and 80 having the size of knapsack 125, 400, 800, and 1,150 respectively. We apply the dynamic programming and GOC algorithm to know the optimal values of the given problem.

**Table 17:** Impact of self-belief-counseling probability for the first problem.

**Self-belief counseling Probability**	**Best**	**Worst**	**Average**	**Median**	**Std. Dev.**
10%	139	124	133	132	3.38
20%	139	124	134	135	2.86
30%	139	126	135	136	2.96
40%	139	131	136	136	2.22
50%	139	131	136	136	2.22
60%	139	131	139	137	2.15
70%	139	130	136	136	2.40
80%	139	127	137	136	2.13
90%	139	125	137	137	2.37

**Table 18:** Impact of self-belief-counseling probability for the second problem.

**Self-belief counseling probability**	**Best**	**Worst**	**Average**	**Median**	**Std. Dev.**
10%	140	130	137	137	2.62
20%	140	135	138	139	2.11
30%	140	135	138	139	2.10
40%	140	135	138	139	2.29
50%	140	135	138	140	2.24
60%	140	135	138	140	2.19
70%	140	135	138	140	2.19
80%	140	135	138	140	2.20
90%	140	135	139	140	1.97

**Table 19:** Impact of self-belief-counseling probability for the third problem.

**Self-belief counseling probability**	**Best**	**Worst**	**Average**	**Median**	**Std. Dev.**
10%	197	167	186	187	6.80
20%	197	167	188	188	5.54
30%	197	176	189	191	4.75
40%	197	173	190	191	4.48
50%	197	175	190	191	4.77
60%	197	182	191	192	3.50
70%	197	175	192	193	3.93
80%	197	182	191	192	3.52
90%	197	178	192	192	3.78

**Table 20:** Impact of self-counseling probability for the first problem.

**Self-counseling probability**	**Best**	**Worst**	**Average**	**Median**	**Std. Dev.**
10%	140	130	137	135	2.53
20%	140	134	138	139	2.18
30%	140	135	138	137	2.21
40%	140	133	138	139	2.12
50%	140	135	138	140	2.17
60%	140	134	138	139	2.08
70%	140	132	138	139	2.33
80%	140	135	138	139	2.22
90%	140	135	138	139	2.26

**Table 21:** Impact of self-counseling probability for the second problem.

**Self-counseling probability**	**Best**	**Worst**	**Average**	**Median**	**Std. Dev.**
10%	139	124	133	133	3.72
20%	139	122	134	134	3.21
30%	139	130	135	135	2.42
40%	139	129	135	136	2.63
50%	139	130	135	136	2.64
60%	139	127	135	136	2.93
70%	139	127	136	136	2.53
80%	139	131	135	136	2.48
90%	139	127	135	136	2.88

**Table 22:** Impact of self-counseling probability for the third problem.

**Self-counseling probability**	**Best**	**Worst**	**Average**	**Median**	**Std. Dev.**
10%	197	163	185	187	6.85
20%	197	172	188	188	5.19
30%	197	175	188	188	5.06
40%	197	172	189	189	4.75
50%	197	175	189	189	4.93
60%	197	176	190	192	3.90
70%	197	178	190	191	4.21
80%	197	178	190	191	3.87
90%	197	175	189	189	4.15

In Table 23, the number of items is 20, having a knapsack size of 125. The optimal value is 261 according to the dynamic programming technique, and the result produced by the GCO is also 261. So the difference between dynamic programming and the proposed algorithm is zero.

**Table 23:** Knapsack problem having 20 items and knapsack size equal to 125.

Items No.	1,2,3,4,5,6,7,8,9,10,11,12,13,14,15,16,17,18,19,20
Weights	1,3,2,5,7,9,12,17,4,6,11,13,15,20,19,18,8,10,14,16
Values	5,12,14,17,19,23,8,16,22,27,21,6,11,10,25,15,20,18,22,13

In Table 24, the number of items is 40, having a knapsack size of 400. The optimal value is 741 according to the dynamic programming technique, and the result produced by the GCO is also 735. So the difference between the dynamic programming and the proposed algorithm is 0.80%.

**Table 24:** Knapsack problem having 40 items and knapsack size equal to 400.

Items No.	1,2,3,4,5,6,7,8,9,10,11,12,13,14,15,16,17,18,19,20,21,22,23,24,25,26,27,28,29,30,31, 32,33,34,35,36,37,38,39,40
Weights	2,4,7,8,10,13,21,5,3,6,12,18,20,1,7,14,22,25,16,24,23,9,11,15,17,27,32,37,39,36,40,35, 19,26,28,38,31,30,29,33
Values	4,7,8,12,13,17,9,19,21,23,27,30,32,18,24,38,37,23,28,41,22,25,34,26,29,15,16,2,1,14, 42,11,20,33,35,39,44,45,50,49

In Table 25, the number of items is 60, having a knapsack size of 800. The optimal value is 951 according to the dynamic programming technique, and the result produced by the GCO is also 945. So the difference between the dynamic programming and the proposed algorithm is 0.63%.

**Table 25:** Knapsack problem having 60 items and knapsack size equal to 800.

Items No.	1,2,3,4,5,6,7,8,9,10,11,12,13,14,15,16,17,18,19,20,21,22,23,24,25,26,27,28,29,30 ,31,32,33,34,35,36,37,38,39,40,41,42,43,44,45,46,47,48,49,50,51,52,53,54,55,56,57 ,58,59,60
Weights	1,7,3,5,2,8,4,9,6,10,12,20,13,18,19,21,30,32,24,33,17,14,12,22,26,31,27,28,34,23, 15,13,2,23,35,40,42,47,50,51,46,49,43,44,55,53,29,4,17,39,24,42,37,34,51,23,12, 17,19,31
Values	18,20,1,7,14,22,25,16,24,23,9,11,15,23,27,30,32,18,24,38,4,7,8,12,13,17,9,19,21, 23,27,30,32,23,3,17,9,19,21,23,27,30,32,18,24,38,19,21,23,27,30,32,18,24,38,37, 23,21,25,12

In Table 26, the number of items is 80, having a knapsack size of 1,150. The optimal value is 1,426 according to dynamic programming techniques, and the result produced by the GCO is also 1,335. So the difference between the dynamic programming and the proposed algorithm is 0.14%.

**Table 26:** Knapsack problem having 80 items and knapsack size equal to 1150.

Items No.	1,2,3,4,5,6,7,8,9,10,11,12,13,14,15,16,17,18,19,20,21,22,23,24,25,26,27,28,29,30,31, 32,33,34,35,36,37,38,39,40,41,42,43,44,45,46,47,48,49,50,51,52,53,54,55,56,57,58, 59,60,61,62,63,64,65,66,67,68,69,70,71,72,73,74,75,76,77,78,79,80
Weights	20,13,18,19,21,30,32,24,33,17,14,12,22,26,31,27,28,34,23,1,7,3,5,2,8,4,9,6,10,12,20, 13,18,19, 47,50,51,46,49,43,44,55, 24,23,9,11,15,17,27,32,37,39,36,40,35,19,26,28, 53,29,4,17,39 ,24,42,37,34,51,23,12,17,28, 18,20,1,7,14,22,25,16
Values	15, 23,27,30,32,18,24,38,7,8,12,13,17,9,19,21,23,27,30,32,23,3,17,11,15,23,27,30,32, 18,24,38,4,7,8,12,13,17,9,19,21,23,27,30,23,27,30,32,18,24,38,37,23,28, 15,16,2,1,14, 42,11,7,14,22,25,16,24,23,9,11,15,23,27,29,35,28,17,28,34

Table 27 shows that the results produced by the GCO algorithms differ by less than 1% by using the dynamic programming method.

**Table 27:** Result of all 0-1 Knapsack problems.

Problem	Optimal value	GCO		Diff (%)
		Z	Time (sec)	
1	261	261	13.48	0
2	741	735	25.0	0.81
3	951	945	35.0	0.63
4	1,426	1335	47.09	0.14

## Conclusion

This article used dynamic programming and the GCO algorithm to solve the 0/1 knapsack problem (KP). GCO is adaptable to the customer’s prerequisites, with various elements, for instance, population size, number of generations, number of counsellors, self-counselling probability, and self-belief counselling probability. The counseling process of each individual continues component-wise in each generation. The GCO algorithm provided other members with counseling and self-counseling ways to influence each individual’s decision. The authors used a third strategy, *i.e.,* self-belief counseling, which illustrates the behavior of an individual’s self-experience in decision-making. The 0/1 knapsack problem is optimally solved with dynamic programming. However, the time complexity of the dynamic programming is O(n3). The proposed GCO algorithm time complexity is O(g ×n ×m)). In this article, we provide a feature analysis of GCO parameters and use it to solve the 0/1 knapsack problem (KP) using GCO. The results show that the GCO is a viable alternative to solving the 0/1 knapsack problem.

##  Supplemental Information

10.7717/peerj-cs.1315/supp-1 Supplemental Information 1 Dataset

10.7717/peerj-cs.1315/supp-2 Supplemental Information 2 Code used for the experiments

## Additional Information and Declarations

### Data Availability

The following information was supplied regarding data availability: The code and data are available in the Supplemental Files.
